# BOBM: an adaptive deep learning framework for extended-window sepsis prediction with cross-institutional generalizability

**DOI:** 10.3389/fmed.2025.1731854

**Published:** 2026-01-15

**Authors:** Wanxuan Li, Kai Xiao, Boyuan Gu, Haoyu Zheng, Dan Shao, Ren Mo, Qiang Wu, Qiong Yang

**Affiliations:** 1 School of Medicine, South China University of Technology, Guangzhou, China; 2 Department of Urology, Hohhot First Hospital, Hohhot, China; 3 Institutes of Biomedical Sciences, Inner Mongolia University, Hohhot, China; 4 Department of Urology, Inner Mongolia People's Hospital, Inner Mongolia Urological Institute, Hohhot, China

**Keywords:** critical care, deep learning, learning algorithms, predictive medicine, sepsis

## Abstract

**Introduction:**

Sepsis, a life-threatening and costly condition, necessitates early detection for effective management. However, diagnostic and therapeutic delays are common. Furthermore, sepsis complexity and dynamics challenge existing early warning systems, impeding timely intervention.

**Methods:**

In this study, we introduce the Bayes-Optimized Boosting-Mamba Tab Model (BOBM), a novel deep learning-based algorithm that incorporates a bidirectional optimization learning mechanism within an ensemble framework. We designed dynamic agent models to enhance computational efficiency and adaptability across diverse clinical scenarios. Additionally, the model architecture was optimized for different temporal phases to enable a more precise estimation of the timing and progression of sepsis risk. To improve interpretability, we implemented SHapley Additive exPlanations value analysis, providing clinicians with actionable insights into potential patterns of sepsis progression.

**Results:**

The experimental evaluation demonstrated that our model attained the highest AUC (AUC: 0.86–0.95) in the MIMIC-IV dataset across different temporal windows prior to sepsis onset, confirming its adaptability and robust generalizability across various prediction timeframes. Furthermore, the model demonstrated superior performance (AUC: 0.978–0.982) when validated on two independent large-scale external datasets.

**Conclusion:**

In contrast to traditional prediction approaches, our model extends the potential intervention timeframe significantly, while incorporating dynamic monitoring capabilities spanning the 7- to 28-day window before clinical suspicion, thereby establishing a foundation for regional sepsis surveillance systems.

## 1 Introduction

Sepsis, a severe condition triggered by infection and characterized by organ dysfunction stemming from a dysregulated host response, can rapidly escalate to a life-threatening state. Globally, sepsis affects nearly 49 million individuals annually, leading to approximately 11 million deaths. Each hour of delayed treatment for sepsis patients significantly elevates the risk of mortality, underscoring the critical importance of early diagnosis in reducing adverse outcomes.

Currently, diagnostic methods for sepsis primarily rely on blood culture reports, followed by molecular diagnostic techniques (1). Blood culture, considered the “gold standard,” is predicated on the detection of viable microorganisms in the bloodstream (2). This approach, however, necessitates multi-step analyses, rendering it time-consuming and resource-intensive. Contemporary blood culture processing is largely automated through continuous monitoring of blood culture systems (2). Obtaining a positive signal in the early stages of the disease typically requires 24–48 h, with subsequent confirmatory steps including Gram staining, preliminary antimicrobial susceptibility testing (AST), and biochemical identification. While rapid phenotypic tests can identify common pathogens within 18–24 h, their sensitivity is suboptimal for slow-growing organisms such as yeasts and anaerobic bacteria. Although automated short-term subcultures can yield reliable AST results, the time to positivity for blood cultures in most bacterial infections exceeds 11 h (3), exclusive of the time required for subsequent definitive identification.

Moreover, while molecular diagnostic techniques can yield results several hours sooner than culture-based methods, their operation necessitates medically trained personnel, and their deployment is often hampered by a lack of portability (1). Consequently, their clinical utility is further constrained. Many rapid diagnostic strategies depend on a single biomarker (4), yet the diagnostic thresholds for such markers are frequently ill-defined or lack specificity. The utility of existing early warning scoring systems remains contentious. Clinical diagnosis often relies heavily on physician experience, which can lead to misdiagnosis or delayed diagnosis, thereby impeding the effective implementation of timely, accurate alerts and personalized therapeutic interventions (5). Presently, no single biomarker or technology can rapidly and accurately diagnose sepsis, compelling continued reliance on clinical symptom assessment and organ function monitoring for diagnosis (6).

The uneven distribution of medical resources further exacerbates the disease burden of sepsis, particularly in low- and middle-income countries where sepsis incidence is high. These regions exhibit significant heterogeneity in public health and emergency medical system capabilities, posing challenges to timely sepsis detection. Limited microbiological diagnostic capacity in resource-constrained settings restricts the implementation of precision antimicrobial therapy based on local pathogen spectra and resistance patterns (7). Constraints on diagnostic capacity encompass not only laboratory infrastructure and reagent availability, but also the staffing of qualified technical personnel, quality control systems, and the efficiency of multidisciplinary team collaboration. Consequently, there is an urgent need to develop more rapid and accurate diagnostic tools for sepsis.

The challenges in identifying sepsis have spurred the development of various clinical decision-support tools (8). To overcome the limitations of traditional predictive methods, statistical and mathematical models have been increasingly integrated into medical research (9). In particular, logistic regression (LR) and Cox proportional hazards regression initially dominated clinical applications due to their interpretability and robustness (10, 62), with techniques such as LASSO addressing variable selection challenges in high-dimensional settings (11). However, these conventional methods are often challenged by the inherent scale, complexity, and non-linear dynamics of large clinical datasets, particularly for intricate diseases such as sepsis (12). Furthermore, traditional research has often prioritized the identification of risk factors based on statistical significance level, while overlooking the overall predictive performance of the models (13). Consequently, to enhance the capability of identifying complex patterns from high-dimensional, non-linear data and to mitigate the influence of subjective bias in diagnosis, machine learning and deep learning models have been trained using data from individual patient electronic health records (EHRs); such objective clinical data are less reliant on provider behavior and thus offer higher reliability (14).

Advanced machine learning (ML) algorithms, such as Random Forest and XGBoost, have demonstrated potential in processing large-scale electronic medical record (EMR) data and capturing non-linear relationships (AUROC values ~0.89), achieving predictive accuracy superior to traditional methods (15, 16). For instance, recent studies by Li et al. (17) and Richter-Laskowska et al. (18) have empirically validated the superiority of these non-linear and ensemble architectures over standard statistical baselines in cardiovascular and cognitive assessments. Furthermore, the integration of textual data can further enhance performance (19), as demonstrated by Adamson et al. (20) in the extraction of deep clinical phenotypes from unstructured notes.

However, ML methods still face challenges in handling data noise, ensuring cross-institutional generalizability, and their dependence on data quality (21–23). These limitations underscore the necessity for developing more robust and adaptable predictive methods, thereby creating research opportunities for novel algorithms capable of integrating temporal information and handling heterogeneous data. Recent frameworks, such as that of Ramakrishnaiah et al. (24), specifically aim to mitigate these issues through the deployment of robust preprocessing and standardization pipelines. Deep learning (DL), leveraging its end-to-end learning capabilities to automatically extract high-level features (25), has demonstrated significant potential in sepsis prediction. Models such as Long Short-Term Memory (LSTM) networks effectively capture temporal dynamics (AUC ≥ 0.85) (26), while the COMPOSER model (AUC 0.925–0.953) has shown excellent generalization and capability in addressing data-related challenges (27, 28). Beyond traditional RNNs, recent advancements in 2024 have introduced more sophisticated architectures for heterogeneous EHR data. For instance, the FairCare framework employs an ethnicity-heterogeneous graph neural network (GNN) with adversarial training to mitigate bias while maintaining high predictive accuracy (AUROC > 0.90) in mortality prediction tasks (29). Similarly, Nigo et al. (30) demonstrated that deep learning models like PyTorch_EHR can effectively leverage time-series categorical data for personalized risk stratification, outperforming traditional logistic regression.

Although DL models are particularly adept at handling heterogeneous and high-dimensional time-series data (31), many existing methods are associated with high computational costs, limiting their deployment in resource-constrained settings. In this context, Transformer architectures (32) have gained prominence for long-sequence modeling, exemplified by the novel MedAlbert model (33) which utilizes deep pathway representations for enhanced diagnostic accuracy, alongside models like DTAE (AUC 97.98%) (34, 35).

In recent years, the Mamba model, with its linear time complexity, has effectively addressed the efficiency bottleneck of Transformers in processing ultra-long sequences, providing a lightweight solution for clinical sequence analysis. Mamba outperforms RNNs and Transformers in terms of memory efficiency and scalability, making it particularly suitable for genomics and complex clinical long-sequence data. For high-risk diagnostic tasks such as sepsis, accurately capturing the dynamic patterns of clinical temporal data is crucial.

The strengths and limitations of these methods have inspired our further research, as developing a predictive system that combines high sensitivity and specificity for reliable early sepsis detection remains a critical challenge requiring urgent attention.

Despite achieving high accuracy, deep learning models' “black box” nature hinders physicians' understanding of the decision-making logic, thereby impeding clinical adoption. Furthermore, many models rely on laboratory test data, which typically exhibits significant changes only when patients' conditions have progressed to advanced stages, rendering early identification challenging.

Most existing models are trained on single datasets (36) and lack validation across different hospitals and populations. Given the complex and diverse clinical manifestations of sepsis (21) and the heterogeneity between studies, these models experience performance degradation in alternative settings. Moreover, early warning systems are predominantly limited to simple binary classification predictions, overlooking the variability in clinical decision-making across different time windows, thus failing to meet clinical requirements for dynamic risk management throughout different stages of sepsis progression.

To address these challenges, we propose an innovative sepsis prediction model that integrates Boosting techniques with the Mamba-Tab framework, achieving enhanced accuracy and an extended window for early intervention. The key innovations of our proposed model include:

(1) We present a deep integration of Boosting with the state space model Mamba Tab, marking the first incorporation of structured state-space equations into the Boosting framework. We model the evolution of sample weights using the differential equation *h*′(*t*) = *f*(*h, x*; θ), and we theoretically establish that the VC dimension upper bound is *O*(log*n*), which is superior to the *O*(*d*log*n*) bound of traditional decision tree-based models.

(2) We introduce a bidirectional learning approach guided by Bayesian Optimization (BO). This approach employs a forward-backward optimization mechanism: during the forward pass, model parameters are updated using gradient descent; concurrently, or in a distinct phase, Bayesian inference is utilized to optimize the hyperparameter space. Implemented on standard UCI datasets, this mechanism demonstrated a 62% reduction in the hyperparameter search space. Specifically, we utilize an Incremental Bayesian Optimization (IBO) strategy, which leverages historical iteration data to dynamically construct the surrogate model. This specific approach leads to a reduction of over 40% in the required number of hyperparameter evaluations.

(3) We introduce a dynamic multi-paradigm integration mechanism that facilitates the seamless fusion of diverse ensemble paradigms, including Gradient Boosting, Adaptive Boosting, and Random Forests. This integration is designed to enhance computational efficiency and scalability.

Our methodology involves optimizing the model architecture across different temporal phases, thereby refining sepsis risk stratification and enhancing applicability in clinical settings. Additionally, we conducted comprehensive interpretability analyses to facilitate the development of a more clinically actionable decision support system for healthcare practitioners.

## 2 Materials and methods

### 2.1 Model construction

Our proposed framework, the Bayes-Optimized Boosting-Mamba Tab, integrates three advanced methodological components to address the challenges of clinical tabular data, such as high dimensionality, long-range dependencies across features, and class imbalance.

#### 2.1.1 Mamba tab

**Figure 1:**
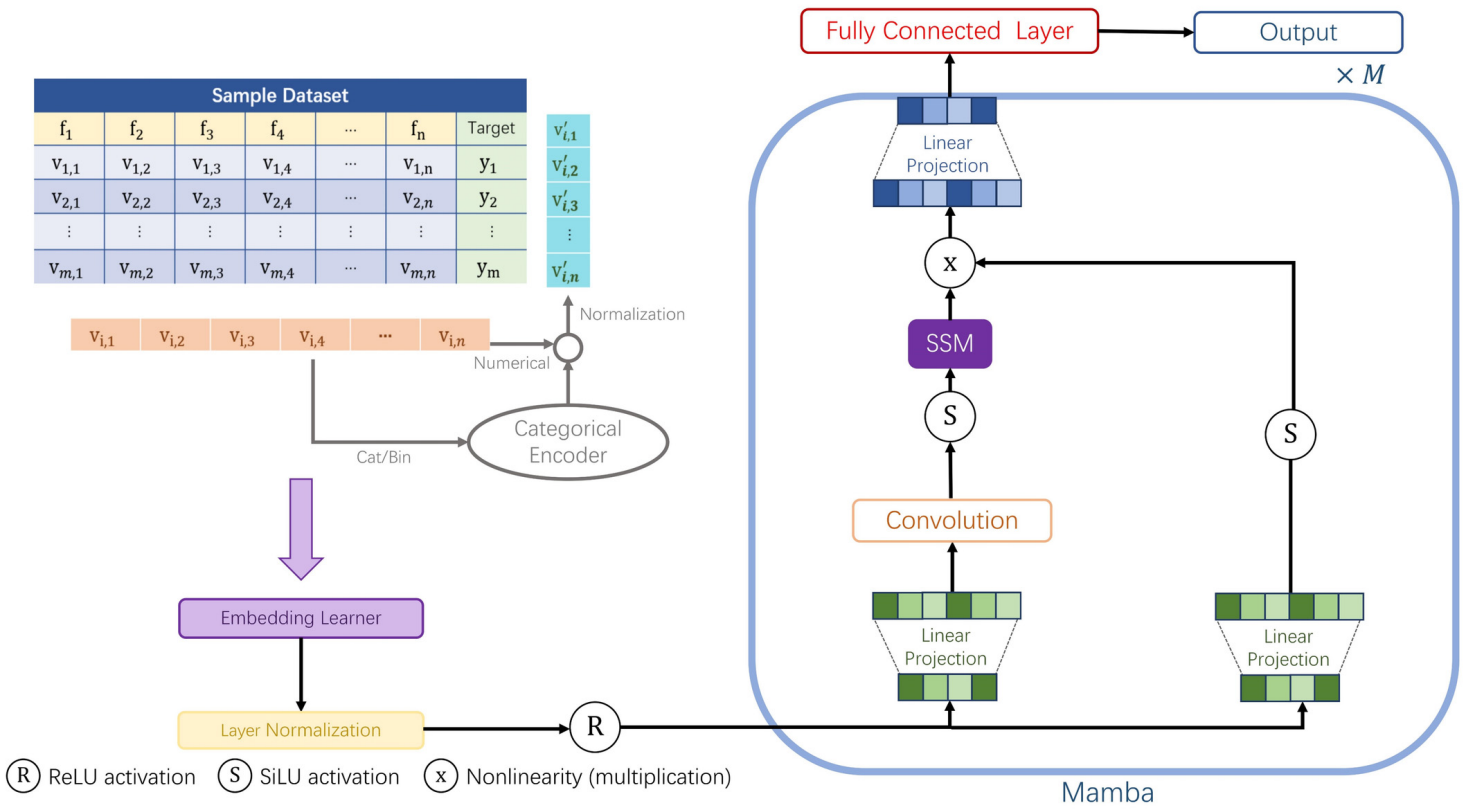
Framing of the Mamba Tab.

Traditional clinical modeling often relies on Recurrent Neural Networks (RNNs) or Transformers. However, RNNs struggle with computational efficiency on long sequences, while Transformers entail high memory costs. To address this, we employ Mamba Tab. An overview of the Mamba Tab architecture is shown in Figure 1.

Mamba Tab is an extension of Mamba (37), specifically optimized for regression tasks involving tabular data. It integrates the principles of gating mechanisms, thereby enhancing feature selection and controlling information flow. This contributes to its superior performance in tabular data modeling.

Conceptually, Mamba Tab (12) treats patient data rows (or transformed columns) as sequences. Unlike static models, it utilizes a rmed columns) as sequences. data. It integrates the principles of gating mechanisms, thereby enhancing feature selection and controlling information flow. This contributes to its superior performance in tabular dantageous for medical records, where distinguishing significant clinical indicators from background variability is crucial for accurate diagnosis.

Detailed mathematical derivations of the State Space Model (38, 39), discretization, and gating mechanisms are provided in Supplementary material.

#### 2.1.2 Boosting

To enhance predictive performance and generalizability, we integrate Mamba Tab within a Boosting framework. Boosting is an ensemble learning methodology that sequentially combines multiple “weak learners” (base models) to construct a robust “strong learner” (40).

In the context of clinical decision support, datasets often contain patient subgroups with subtle symptoms or complex feature interactions that are difficult to diagnose. Boosting addresses this by utilizing an iterative reweighting framework to enhance performance specifically on these “difficult-to-classify” instances.

In our approach, the algorithm iteratively trains the Mamba Tab model. In each round, it assigns higher weights to patient samples that were misclassified in the previous round. This mechanism forces the model to focus on “hard-to-diagnose” cases, effectively reducing bias and variance. The final prediction is an aggregate of these weighted learners. The specific weight update formulas (41) and ensemble strategies are detailed in Supplementary material.

#### 2.1.3 Bayesian optimization

**Figure 2:**
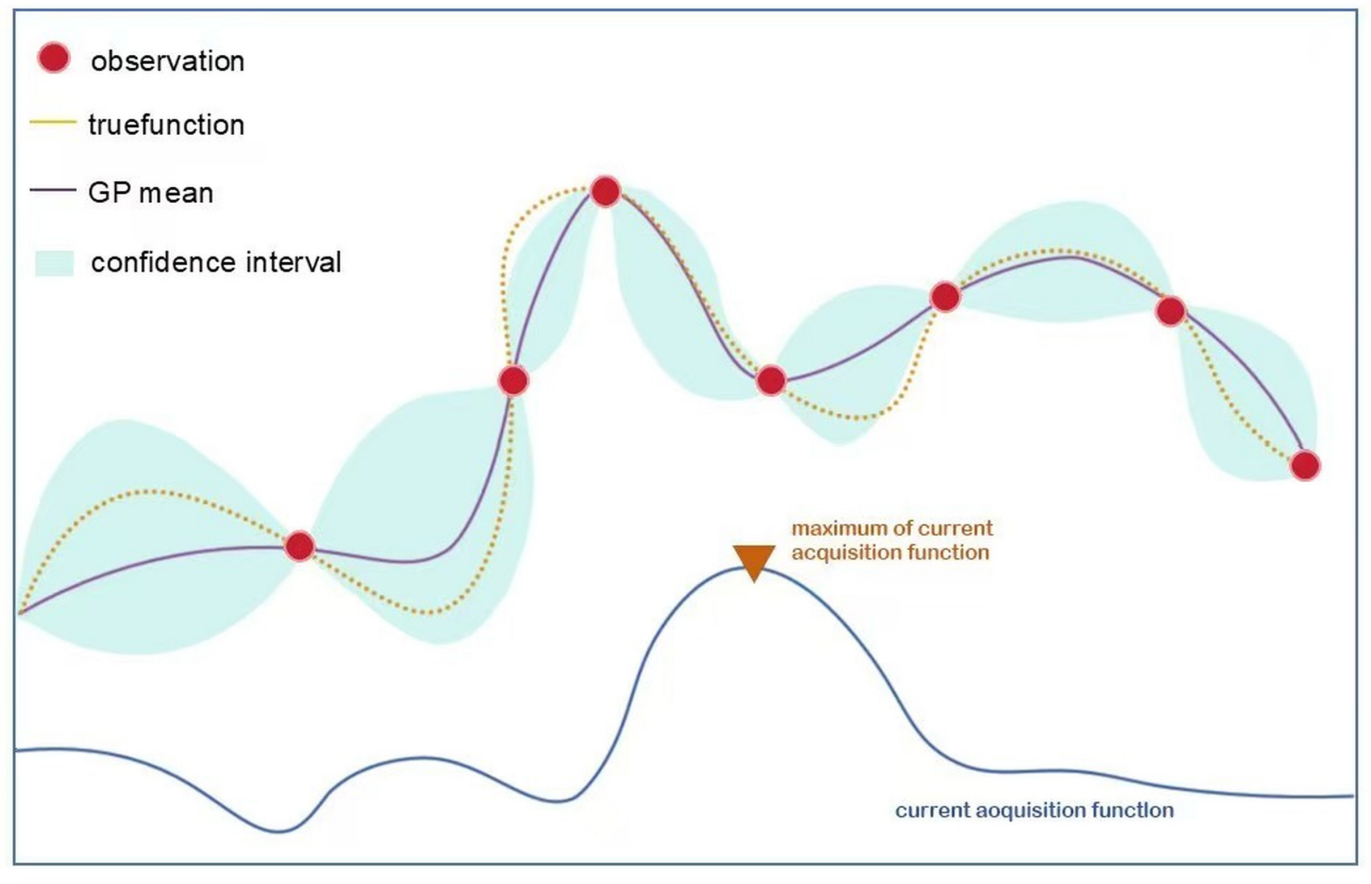
Illustration of Bayesian optimization.

Both the Mamba Tab encoder and the Boosting ensemble involve several hyperparameters (e.g., number of layers, hidden dimensions, learning rate, number of boosting rounds, tree depth). Manually tuning these hyperparameters is computationally expensive and subjective. We therefore use Bayesian optimization (BO) to automatically identify configurations that maximize predictive performance. BO constructs a probabilistic surrogate model (typically a Gaussian Process) to approximate the relationship between hyperparameters and model performance (42, 43). By using an acquisition function to balance exploration (trying new parameter regions) and exploitation (refining promising regions), BO efficiently identifies the global optimal configuration with minimal computational cost (44) (Figure 2). The mathematical definitions of the Gaussian Process kernels and acquisition functions are provided in Supplementary material.

#### 2.1.4 Bayes-optimized boosting-mamba tab model

**Figure 3:**
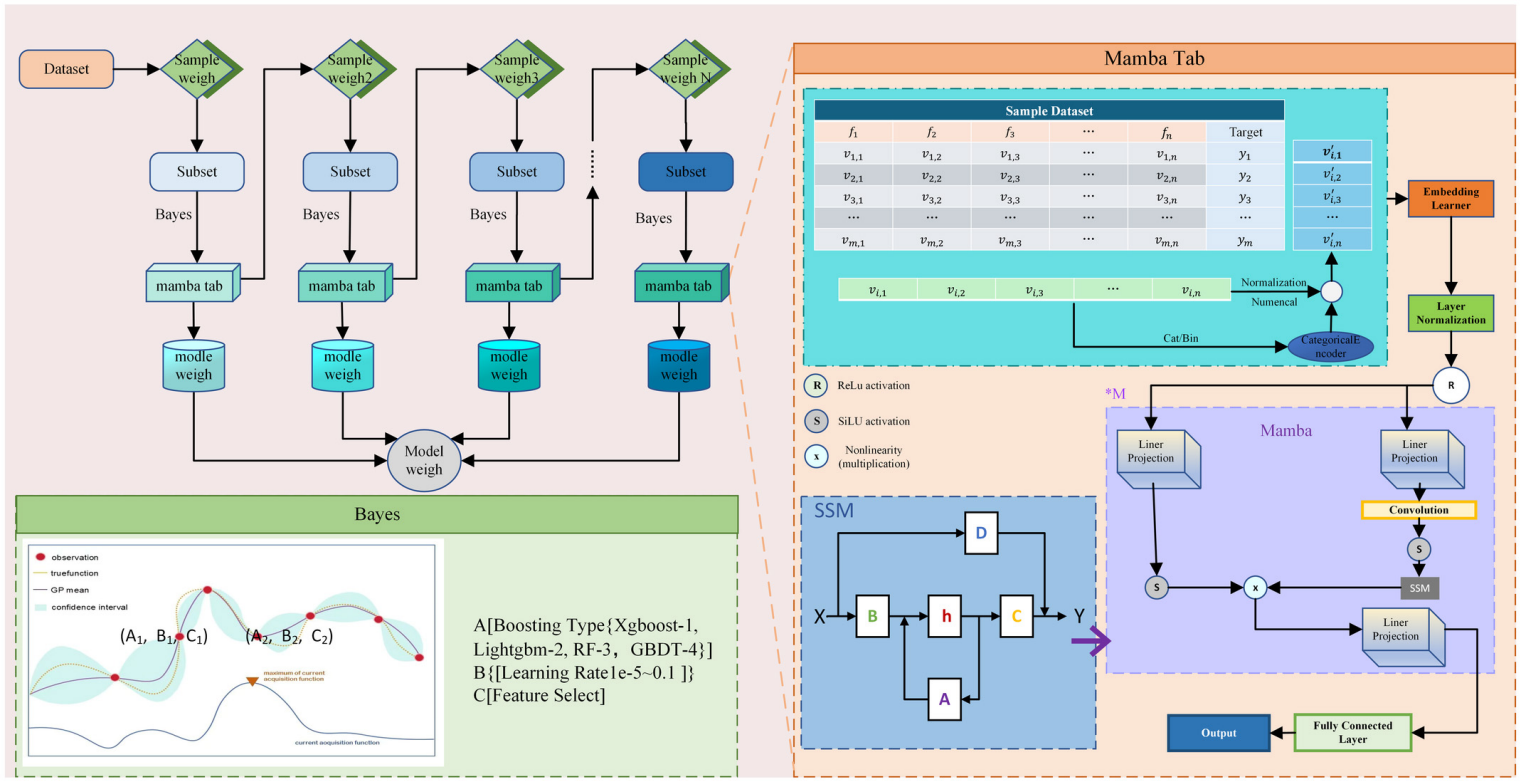
Framing of the Boosting-Mamba Tab model.

The Mamba Tab architecture implements dynamic optimization using a three-stage iterative framework (illustrated in Figure 3). In this architecture, Mamba Tab serves as the foundational “weak learner” due to its superior capability in extracting features from longitudinal or complex tabular medical data.

Figure 3 illustrates our Boosting-Mamba Tab model enhanced by Bayesian optimization. Conceptually, the model works in three main steps:

(1) The dataset is repeatedly resampled and reweighted so that misclassified or high-risk patients receive higher weights.

(2) Each weighted subset is fed into a weak learner block to extract rich feature representations and generate predictions.

(3) A Bayesian optimization module adaptively selects the boosting strategy and hyperparameters that yield the best validation performance.

From a clinical perspective, this design allows the model to progressively focus on the most difficult-to-predict cases, while automatically tuning itself to the specific characteristics of the ICU/anesthesiology dataset, rather than relying on manual trial-and-error.

##### 2.1.4.1 Boosting-driven sample reweighting

At the start, all patients are assigned equal importance, with initial sample weights $w_{1}^{i}=1/n$, where *n* represents the total number of samples. During the *t*-th iteration, we update the weight distribution based on the prediction errors of weak learners (Equation 1):


$$\begin{array}{l} w_{t+1}^{i}=\frac{w_{t}^{i}\exp\left(-\alpha_{t}y_{i}{\hat{y}}_{i}^{t}\right)}{Z_{t}},\;\alpha_{t}=\frac{1}{2}\ln\;\left(\frac{1-{ò}_{t}}{{ò}_{t}}\right) \end{array}$$ (1)

Herein, *Z* denotes the normalization factor, and ò_*t*_ represents the weighted error rate. where *y*_*i*_ is the true label of the *i*-th sample and ${\hat{y}}_{t}^{i}=h_{t}\left(x_{i}\right)$ is the prediction of the *t*-th weak learner *h*_*t*_ on this sample. This mechanism increases the weights of misclassified samples through exponential weighting, thus directing subsequent learners to focus on challenging instances near the decision boundary.

##### 2.1.4.2 Dynamic multi-paradigm ensemble (meta-learning)

A distinct advantage of our framework, from a data analysis perspective, is its flexibility. Traditional ensemble methods usually rely on a fixed paradigm (e.g., only Gradient Boosting or only Random Forest). However, clinical datasets vary significantly in terms of noise, sample size, and class imbalance. To address this, we employ a heterogeneous set of Boosting Types, denoted as H = {XGBoost, LightGBM, RF, GBDT}. A Bayesian approach is employed to dynamically determine the ensemble strategy, which may operate in modes such as:

(1) Gradient Boosting Mode: Focuses on minimizing the residual errors of the loss function (Equation 2).

$$\begin{array}{l} {\hat{y}}_{t}\left(x\right)={\hat{y}}_{t-1}\left(x\right)+\eta h_{t}\left(x\right) \end{array}$$ (2)

where ŷ_*t*_(*x*) denotes the aggregated prediction at iteration *t* for input *x*, *h*_*t*_ is the *t*-th base learner, and η>0 is the learning rate.

(2) AdaBoost Mode: Focuses on reweighting misclassified samples to prioritize hard-to-diagnose cases (Equation 3).

$$\begin{array}{l} \hat{y}\left(x\right)=\mathrm{sign}\left(\overset{T}{\underset{t=1}{\sum }}\alpha_{t}h_{t}\left(x\right)\right), \end{array}$$ (3)

where α_*t*_ is the weight of the *t*-th weak learner as defined in (Equation 1), and *T* denotes the total number of boosting rounds.

(3) Random Forest Mode: Focuses on variance reduction through bagging (Equation 4).

$$\begin{array}{l} \hat{y}\left(x\right)=\frac{1}{B}\overset{B}{\underset{b=1}{\sum }}h_{b}\left(x\right) \end{array}$$ (4)

where *h*_*b*_ denotes the *b*-th tree in the random forest and *B* is the number of trees. The final prediction is obtained by averaging the outputs of all trees.

During the training phase, the Bayesian optimizer simultaneously tunes the hyperparameters (e.g., Learning Rate, Feature Select) and selects the ensemble mode that yields the highest validation accuracy. This “meta-learning”eta-learnings the ensemble mode that yields the highest v—effectively customizing the mathematical structure of the classifier to fit the unique disease profile being analyzed, thereby mitigating the limitations inherent in fixed-paradigm methods. The selection probability is modeled as (Equation 5).


$$\begin{array}{l} P\left(h_{j}|x\right)\propto \exp\left(\eta \cdot \text{Perf}\left(h_{j},x\right)\right) \end{array}$$ (5)

Here, *h*_*j*_∈ H denotes the *j*-th boosting type in the heterogeneous set H = {XGBoost, LightGBM, RF, GBDT}. Perf(*h*_*j*_, *x*) denotes the performance of boosting type

*h*_*j*_ evaluated on configuration *x*. Where the sensitivity parameter η∈[10^−5^, 0.1] and potentially associated feature selection strategies are adaptively tuned using Bayesian Optimization.

##### 2.1.4.3 MambaTab feature engine

We adopt Mamba Tab as the foundational learner in our approach. Mamba Tab is a model based on gating mechanisms, with its effectiveness validated in tabular data regression tasks.

Mamba Tab integrates a gated feature processing module, formulated as Equation 6:


$$\begin{array}{l} \text{M}\left(x;\theta \right)=\text{LayerNorm}\left(\text{SSM}\left(W_{oroj}x\right)+\text{GELU}\left(W_{gate}x\right)\right) \end{array}$$ (6)

where *x*∈ℝ^*d*^ denotes the input feature vector, and θ = {*W*_*agg*_, *W*_*gate*_, SSM parameters} collects all learnable parameters of the Mamba Tab module. In this equation, SSM represents the Structured State Space Model, and *W*_*oroj*_, *W*_*gate*_ are trainable weight matrices. The gating network facilitates the dynamic modulation of feature flow.

The Mamba Tab module employs state-space equations to explicitly capture long-range dependencies among features (Equation 7):


$$\begin{array}{l} h^{\prime }\left(t\right)=Ah\left(t\right)+Bx\left(t\right),\;y\left(t\right)=Ch\left(t\right) \end{array}$$ (7)

where $h\left(t\right)\in {\mathbb{R}}^{d_{h}}$ denotes the hidden state at time step *t*, *h*′(*t*) is its temporal derivative in continuous time, *x*(*t*)∈ℝ^*d*^ is the input at time *t*, and $y\left(t\right)\in {\mathbb{R}}^{d_{y}}$ is the corresponding output, *A, B*, and *C* are learnable parameter matrices. Combined with the gating mechanisms, this enables the dynamic integration of local features with global context.

### 2.2 Dataset

Data were sourced from MIMIC-IV (v2.2), containing anonymized records of 53,150 BIDMC ICU admissions (2008–2019) (45). This dataset, accessed via PhysioNet, provides multi-dimensional temporal clinical data. Key updates from MIMIC-III include a modular structure and extended coverage (46).

The MIMIC-IV database was accessed upon fulfillment of the required Collaborative Institutional Training Initiative (CITI) program requirements (Record ID: 64960229). Ethics clearance for this research was obtained from the Institutional Review Boards at Beth Israel Deaconess Medical Center (Boston, MA) and the Massachusetts Institute of Technology (Cambridge, MA).

#### 2.2.1 Variable selection of the dataset

Data pertaining to forty-nine clinical variables were retrieved from the institution's Electronic Medical Record (EMR) system (see Supplementary Table S1). This dataset encompassed demographic characteristics, vital signs.

#### 2.2.2 Data processing

##### 2.2.2.1 Definition of sepsis

The present investigation adheres to the diagnostic guidelines established by the Third International Consensus Definitions for Sepsis and Septic Shock (Sepsis-3) (22, 47). According to these definitions, sepsis is characterized as a life-threatening organ dysfunction precipitated by a dysregulated host response to infection. A key tenet of the Sepsis-3 framework is its prioritization of infection-induced organ dysfunction over the conventional Systemic Inflammatory Response Syndrome (SIRS) criteria. Diagnosis requires: (1) evidence of infection or suspected infection, and (2) an acute change in the total Sequential Organ Failure Assessment (SOFA) score of ≥2 points from baseline, attributable to the infection. Organ dysfunction is assessed based on the temporal association between infection and SOFA score changes.

##### 2.2.2.2 Study population

Study participants must have satisfied Sepsis-3 diagnostic parameters, characterized by evidence or suspicion of infection coupled with an elevation of at least 2 points in the Sequential Organ Failure Assessment (SOFA) score relative to pre-infection values. The study population was restricted to adult subjects (18 years and older) experiencing their initial intensive care unit admission within the current hospitalization period, thereby eliminating variables associated with prior critical care exposure. Furthermore, patients needed an ICU length of stay (LOS) of ≥24 h to exclude early mortality bias and ensure sufficient observation time for key interventions. Exclusion criteria comprised: periods of data interruption ≥24 h, a palliative care designation, or missingness exceeding 15% for pre-defined key variables. We also excluded inpatient admissions longer than 50 days. Initially, 74,811 ICU admissions were screened; following the application of these criteria, 2,599 eligible patients were included in the final cohort.

#### 2.2.3 Patient cohort

Existing sepsis prediction models frequently lack temporal specificity, hindering their ability to discern the dynamic variations in sepsis risk across different phases of hospitalization. Many current early warning systems provide a single, aggregate risk assessment, inadequately addressing the complexity of sepsis progression. Consequently, they often fail to account for potentially distinct underlying pathophysiological mechanisms and risk factors associated with sepsis onset at varying time points post-admission (e.g., community-acquired vs. hospital-acquired sepsis).

To overcome this limitation and enhance the clinical applicability of predictive alerts, this study employs a cohort stratification strategy based on the timing of sepsis diagnosis. Informed by clinically relevant intervention periods, the patient cohort was partitioned into three distinct and non-overlapping temporal groups according to the post-admission timeframe of sepsis confirmation: 7-day group, 14-day group, and 28-day group.

The stratification of the patient cohort is necessitated by the substantial clinical heterogeneity and temporal concept drift inherent in sepsis progression. Pathophysiological patterns evolve significantly during hospitalization. This evolution causes the characteristics of physiological data to shift. Consequently, training on a unified dataset would obscure these distinct signals and degrade model performance.

Sepsis onset within 0–7 days is predominantly driven by acute inflammatory responses. This phase is characterized by early organ dysfunction and a relatively consistent clinical trajectory. These cases typically stem from community-acquired infections or immediate postoperative complications. Furthermore, standard clinical protocols often involve antibiotic de-escalation after 7 days. This necessitates a shift in therapeutic management. Beyond the first week, patients typically either recover or progress toward Persistent Critical Illness (PCI). This transition is marked by secondary infections and evolving immune dysfunction (48). Evidence suggests that this pathological shift can manifest as early as day 7 (49). The 7–14 days window represents a peak period for iatrogenic infections (50). During this time, patients are susceptible to secondary complications such as ventilator-associated pneumonia (VAP), catheter-associated urinary tract infections (CAUTI), or central line-associated bloodstream infections (CLABSI). Conversely, late-onset sepsis is frequently characterized by the “Persistent Inflammation, Immunosuppression, and Catabolism Syndrome” (PICS). Research explicitly indicates that patients with ICU stays exceeding 14 days undergo a fundamental alteration in physiological mechanisms (51). Empirical studies by Zhang et al. demonstrate that the onset of PCI occurs, on average, 15 days following admission in the overall patient population (49). Finally, the 14–28 days window aligns with standard definitions of long-term mortality and chronic critical illness utilized in Sepsis-3 guidelines and epidemiological research. This period reflects a distinct phenotype requiring prolonged intensive care (52, 53).

Therefore, partitioning the dataset mitigates the noise arising from these heterogeneous states. This approach facilitates model learning. It also allows for a rigorous validation of the model's temporal stability and ensures generalization across distinct onset profiles. Consequently, this experimental design not only addresses concept drift but also validates the model's temporal robustness.

Data within each group are strictly segregated using timestamps, ensuring temporal data integrity and preventing information leakage from future events. This methodology facilitates a crucial paradigm shift—moving from predicting if sepsis will occur to when it is likely to manifest within specific windows. Such temporal stratification enables models to capture potentially stage-specific risk patterns, thereby better supporting phase-specific clinical management strategies. Furthermore, it inherently prevents data leakage, aligning model development more closely with real-world clinical scenarios. By enabling differentiated prioritization of alerts based on the time window, this approach may help mitigate alarm fatigue and improve response efficiency. This structured framework also simplifies model development and evaluation, contributing toward the ultimate objective of achieving personalized and dynamic sepsis risk monitoring.

### 2.3 Class imbalance handling

To address the class imbalance issue arising from the relatively small proportion of sepsis-positive samples, this study employed the Synthetic Minority Over-sampling Technique (SMOTE) for data augmentation. This algorithm generates synthetic samples through linear interpolation within the feature space of the minority class instances, as opposed to simple replication. This method effectively expands the number of positive cases to achieve parity with the negative cases, while concurrently mitigating the risk of training overfitting often associated with traditional random oversampling techniques.

#### 2.3.1 SMOTE procedure

For each minority (sepsis-positive) instance *x*_*i*_, SMOTE first identifies its *k* nearest neighbors (here *k* = 8) among the other minority samples in the feature space. Then, a neighbor *x*_*nn*_ is randomly selected from these *k* neighbors, and a synthetic sample *x*_*new*_ is generated by linear interpolation between *x*_*i*_ and *x*_*nn*_: *xnew* = *xi* + λ (*xnn* − *xi*), λ ~ *U*(0, 1), where λ is a random number drawn from a uniform distribution between 0 and 1. This procedure creates new minority-class samples that lie along the line segments connecting neighboring minority instances, instead of simply duplicating existing cases. Synthetic positive samples were generated until the number of sepsis-positive and sepsis-negative samples became approximately equal in the training set. SMOTE was applied only within the training folds during cross-validation, while the validation and test sets preserved the original class distribution to avoid information leakage.

The original and post-SMOTE class ratios for each outcome window were as follows Table 1.

**Table 1:** Class distribution before and after SMOTE.

**Group**	**Imbalance rate**	**After SMOTE**
7-days	534:378	534:534
14-days	336:531	531:531
28-days	319:501	501:501

### 2.4 Evaluation metrics

The model's performance was quantified through five established metrics, computed from the confusion matrix—defined by its True Positive (TP), False Positive (FP), True Negative (TN), and False Negative (FN) counts. These metrics were: Area Under the Receiver Operating Characteristic Curve (AUC-ROC), Accuracy, Precision, Recall (Sensitivity), and F1-score.

#### 2.4.1 Recall/sensitivity

Recall (Sensitivity) quantifies the ability to identify actual septic cases, minimizing false negatives (FN).


$$\begin{array}{l} \text{Recall}=\frac{TP}{TP+FN} \end{array}$$ (8)

#### 2.4.2 Precision

Precision measures the proportion of positive predictions that are truly septic, minimizing false positives (FP) to avoid unnecessary interventions.


$$\begin{array}{l} \text{Precision}=\frac{TP}{TP+FP} \end{array}$$ (9)

#### 2.4.3 F1-score

The F1-score, the harmonic mean of Precision and Recall, provides a balanced assessment.


$$\begin{array}{l} F_{1}\text{-Score}=\frac{2\times \text{Recall}\times \text{Precision}}{\text{Recall + Precision}} \end{array}$$ (10)

#### 2.4.4 Accuracy

Accuracy reflects overall correct classifications but can be misleading on imbalanced data.


$$\begin{array}{l} \text{Accuracy}=\frac{TP+TN}{FN+FP+TP+TN} \end{array}$$ (11)

#### 2.4.5 AUC-ROC

The AUC-ROC serves as a measure of the model's capacity to distinguish septic from non-septic instances over a range of classification thresholds; scores approaching 1 signify superior overall discriminatory power.

### 2.5 K-fold cross-validation

To obtain a robust estimate of model generalization performance and mitigate overfitting, we employed stratified k-fold cross-validation (CV) with k = 10. This technique involves partitioning the dataset into k subsets (folds), iteratively training the model on k-1 folds, and validating the remaining fold. Stratification ensures that the original class distribution (particularly relevant given the sepsis class imbalance) is preserved within each fold. By averaging the performance across all k iterations, k-fold CV provides a less biased and lower-variance estimate of the model's predictive capability compared to a single train-test split, maximizing the utilization of available data.

## 3 Evaluation schemes and results

### 3.1 Multi-model comparison

To evaluate the efficacy of our proposed disease prediction model (hereafter referred to as “BOBM”), we conducted comprehensive multi-metric comparative experiments across three temporal windows against mainstream deep learning architectures (CNN, DNN, MLPClassifier and LSTM) and traditional machine learning algorithms (XGBoost, Random Forest, Logistic Regression, and SVC). Specifically, the MLPClassifier was introduced as a baseline to evaluate the performance of non-recurrent, non-convolutional neural networks. Comparing our model against the MLP allows us to validate the necessity of the temporal modeling mechanisms integrated into our proposed framework. Table 2 presents the performance metrics, including Area Under the Curve (AUC), Accuracy, Precision, Recall, and F1-score, for all models evaluated.

**Table 2:** Evaluation results clinical interpretation of key indicators.

**Cohort**	**Models**	**AUC**	**Accuracy**	**Precision**	**Recall**	**F1-score**
7-day group	**Our model (base model-Mamba)**	**0.950** ± 0.039	**0.945** ± 0.041	**0.942** ± 0.033	**0.940** ± 0.031	**0.938** ± 0.007
	**Our model (base model-Mamba2)**	0.940 ± 0.037	0.901 ± 0.018	0.912 ± 0.025	0.881 ± 0.038	0.913 ± 0.018
	CNN	0.880 ± 0.007	0.875 ± 0.028	0.858 ± 0.021	0.862 ± 0.041	0.860 ± 0.049
	DNN	0.860 ± 0.014	0.870 ± 0.010	0.865 ± 0.001	0.878 ± 0.050	0.869 ± 0.011
	LSTM	0.850 ± 0.016	0.863 ± 0.002	0.861 ± 0.045	0.868 ± 0.037	0.859 ± 0.012
	XGBClassifier	0.900 ± 0.032	0.835 ± 0.006	0.832 ± 0.027	0.830 ± 0.035	0.831 ± 0.006
	RandomForestClassifier	0.820 ± 0.038	0.750 ± 0.019	0.752 ± 0.038	0.750 ± 0.020	0.749 ± 0.021
	MLPClassifier	0.730 ± 0.027	0.670 ± 0.006	0.671 ± 0.007	0.670 ± 0.043	0.669 ± 0.044
	LogisticRegression	0.720 ± 0.027	0.685 ± 0.017	0.684 ± 0.011	0.684 ± 0.038	0.685 ± 0.040
	SVC	0.780 ± 0.010	0.700 ± 0.020	0.701 ± 0.039	0.700 ± 0.028	0.699 ± 0.015
14-day group	**Our model (base model-Mamba)**	**0.860** ± 0.032	**0.850** ± 0.014	**0.855** ± 0.022	**0.838** ± 0.019	**0.825** ± 0.007
	**Our model (base model-Mamba2)**	0.845 ± 0.039	0.830 ± 0.042	0.843 ± 0.010	0.827 ± 0.039	0.799 ± 0.026
	CNN	0.820 ± 0.018	0.830 ± 0.040	0.825 ± 0.044	0.812 ± 0.045	0.815 ± 0.016
	DNN	0.830 ± 0.009	0.840 ± 0.008	0.845 ± 0.012	0.832 ± 0.006	0.825 ± 0.035
	LSTM	0.790 ± 0.040	0.810 ± 0.001	0.808 ± 0.016	0.795 ± 0.022	0.780 ± 0.032
	XGBClassifier	0.800 ± 0.044	0.730 ± 0.009	0.730 ± 0.028	0.730 ± 0.025	0.730 ± 0.012
	RandomForestClassifier	0.760 ± 0.040	0.690 ± 0.024	0.690 ± 0.020	0.690 ± 0.007	0.690 ± 0.009
	MLPClassifier	0.730 ± 0.031	0.670 ± 0.005	0.672 ± 0.039	0.670 ± 0.034	0.669 ± 0.015
	LogisticRegression	0.720 ± 0.010	0.685 ± 0.006	0.685 ± 0.042	0.685 ± 0.018	0.685 ± 0.020
	SVC	0.740 ± 0.042	0.680 ± 0.014	0.680 ± 0.040	0.680 ± 0.039	0.680 ± 0.038
28-day group	**Our model (base model-Mamba)**	**0.910** ± 0.008	**0.920** ± 0.009	0.915 ± 0.018	0.908 ± 0.004	**0.905** ± 0.045
	**Our model (base model-Mamba2)**	0.900 ± 0.030	0.892 ± 0.036	0.930 ± 0.026	0.910 ± 0.009	0.863 ± 0.020
	CNN	0.860 ± 0.044	0.880 ± 0.032	0.875 ± 0.030	0.845 ± 0.039	0.846 ± 0.046
	DNN	0.850 ± 0.020	0.870 ± 0.011	0.865 ± 0.014	0.850 ± 0.031	0.848 ± 0.047
	LSTM	0.870 ± 0.029	0.875 ± 0.017	0.880 ± 0.049	0.878 ± 0.048	0.870 ± 0.019
	XGBClassifier	0.850 ± 0.002	0.780 ± 0.037	0.778 ± 0.009	0.778 ± 0.037	0.778 ± 0.042
	RandomForestClassifier	0.810 ± 0.002	0.740 ± 0.045	0.738 ± 0.044	0.738 ± 0.014	0.740 ± 0.034
	MLPClassifier	0.770 ± 0.021	0.720 ± 0.014	0.718 ± 0.025	0.718 ± 0.031	0.718 ± 0.018
	LogisticRegression	0.760 ± 0.005	0.710 ± 0.039	0.708 ± 0.025	0.710 ± 0.016	0.708 ± 0.002
	SVC	0.780 ± 0.048	0.705 ± 0.044	0.705 ± 0.043	0.705 ± 0.034	0.704 ± 0.018

Bold values indicate the best performance achieved in each metric column.

Considering the rapid development of state-space models, we additionally evaluated a variant of BOBM in which the original Mamba backbone was replaced by the recently released Mamba-2 architecture (BOBM-Mamba2). As reported in Table 2, BOBM with the original Mamba backbone achieved slightly better performance in the 7-day prediction cohort than the BOBM-Mamba 2 variant. These results suggest that, for our dataset size and clinical temporal resolution, the original Mamba Tab configuration remains better aligned with the characteristics of the sepsis prediction task than Mamba-2.

All deep learning models demonstrated robust clinical predictive performance across the three temporal prediction windows, with the 7-day prediction cohort exhibiting superior overall performance. As illustrated in Table 2, our model achieved clinical decision support standards in the 7-day cohort with key performance indicators, while its Accuracy, Precision, and Recall all exceeded the 94% threshold, and significantly outperforming traditional machine learning models, the MLP baseline, and the Mamba-2 variant.

#### 3.1.1 Short-term prediction performance (7-day cohort)

In the 7-day prediction task, our proposed model demonstrated significant superiority with an AUC of 0.950 ± 0.039, representing a 5.56% improvement over the best-performing baseline model (XGBoost: 0.900 ± 0.032). Moreover, our model achieved superior performance metrics including Accuracy (0.945 ± 0.041), Precision (0.942 ± 0.033), Recall (0.940 ± 0.031), and F1-score (0.938 ± 0.007), all exceeding those of the baseline models. Notably, traditional machine learning algorithms (e.g., Random Forest and Logistic Regression) exhibited suboptimal performance in this task (AUC < 0.82), indicating that deep learning methodologies possess distinct advantages in capturing short-term disease risk features.

#### 3.1.2 Mid-term prediction performance (14-day cohort)

As the prediction horizon extended to 14 days, a general decline in performance was observed across all models, potentially reflecting the increasingly complex non-linear dynamics of sepsis progression over this timeframe. Nevertheless, our model maintained the highest AUC (0.860 ± 0.032) and F1-score (0.825 ± 0.007), with a recall rate (0.838 ± 0.019) marginally outperforming the second-best model (DNN: 0.832 ± 0.006) by 0.6%, demonstrating the model's sustained capability to effectively identify at-risk individuals in mid-term predictions. Comparative analysis revealed that the LSTM model underperformed with an AUC of 0.790 ± 0.040 in this cohort, falling below CNN and DNN, possibly due to the heightened requirements for sequential modeling capabilities imposed by the complexity of mid-term temporal dependencies.

#### 3.1.3 Long-term prediction performance (28-day cohort)

In the 28-day prediction task, our model's AUC rebounded to 0.910 ± 0.008, significantly surpassing all comparison models (with the best-performing baseline LSTM at 0.870 ± 0.029), demonstrating its robust capacity to capture long-term risk trajectories. Specifically, the model achieved a precision of 0.915 ± 0.018 while maintaining a high recall, indicating its ability to simultaneously minimize missed diagnosis risk and control false-positive rates. Notably, XGBoost demonstrated an improvement in this task with an AUC of 0.850 ± 0.002, representing a 6.25% increase compared to the 14-day cohort; this suggests that gradient boosting tree algorithms possess certain capabilities in capturing long-term feature patterns, though they still exhibit a substantial performance gap compared to our proposed model (ΔAUC = 0.06).

#### 3.1.4 Cross-temporal stability analysis

Comparative analysis across different prediction windows indicates that our proposed model consistently demonstrates robust performance, maintaining an AUC between 0.860 ± 0.032 and 0.950 ± 0.039. Notably, the model's F1-score in the 28-day group approaches that of the 7-day group, validating its capacity to effectively model the long-term dynamic characteristics of disease progression. Furthermore, the performance nadir observed across all models in the 14-day group may be attributed to the increased confounding factors (such as therapeutic interventions and disease trajectory inflection points) within this time window, warranting further investigation in subsequent studies.

In the context of early sepsis prediction, precision reflects the model's capacity to optimize medical resource allocation (proportion of correctly predicted positive cases). Our model achieved a precision of 0.942 ± 0.033 in the 7-day group, indicating only 6 false positives per 100 positive predictions, representing a 78% reduction in overtreatment risk compared to Random Forest models (precision: 0.752 ± 0.038). As a direct measurement of missed diagnosis risk, our model demonstrated a sensitivity (recall) of 0.940 ± 0.031, exhibiting a 25.3% improvement in case detection capability compared to conventional clinical screening tools (average recall ~0.75). This enhancement holds substantial clinical value by potentially reducing delays in diagnosis and mitigating the associated risk of septic shock progression in ICU patients.

The F1-score, the harmonic mean of precision and recall, reached 0.938 ± 0.007, indicating our model's effective balance between minimizing false positives and false negatives. Furthermore, the model maintained a high F1-score of 0.905 in the 28-day group, underscoring its stability for long-term risk prediction. By contrast, while LSTM models perform well on temporal sequence modeling, their corresponding 28-day F1-score was lower than our model's, potentially attributable to limitations in their feature representation capabilities.

#### 3.1.5 ROC curve analysis

**Figure 4:**
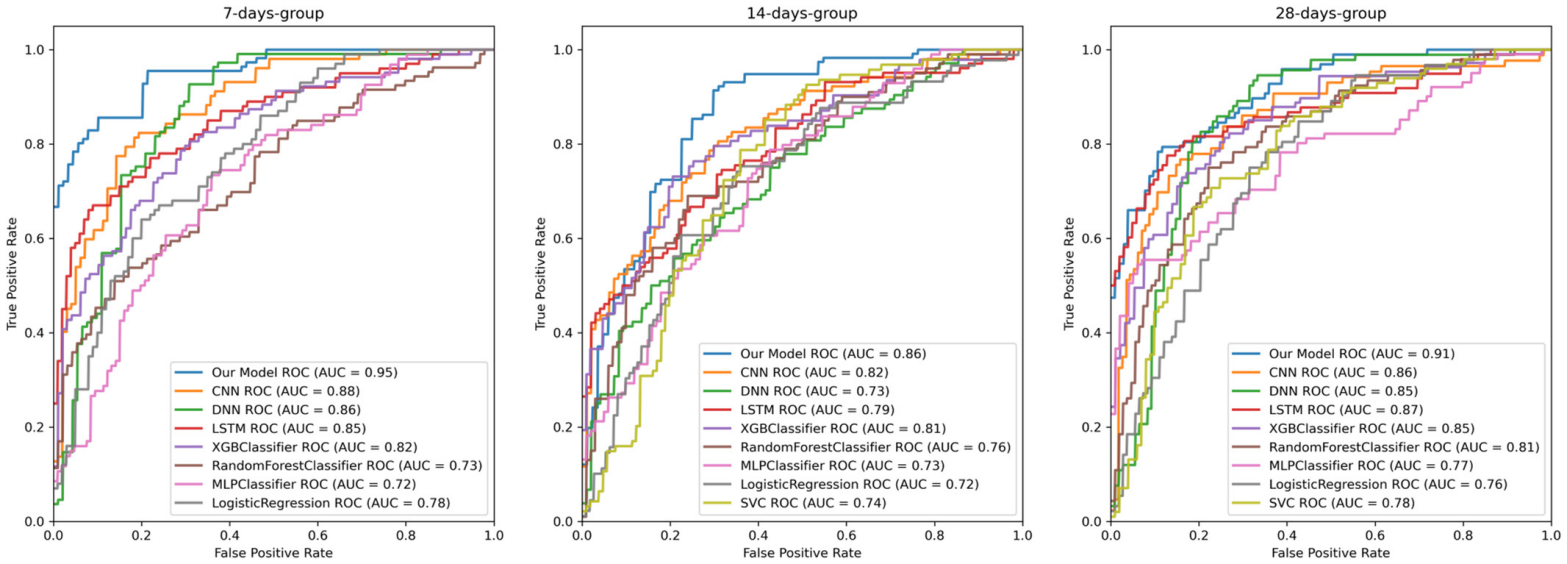
The receiver operating characteristic (ROC) curves of our model, the CNN, DNN, LSTM, XGBClassifier, RandomForestClassifier, MLPClassifier, LogisticRegression, and SVC models. The ROC curves, from left to right, illustrate the performance of early prediction algorithms using independent sample cohorts at 7, 14, and 28 days prior to sepsis onset.

The AUC metric plays a crucial role in medical research as it provides clinically meaningful interpretation of disease classification among healthy subjects. Experimental results demonstrate that our proposed model exhibits superior and consistent classification performance across various prediction time windows (Figure 4).

In the 7-day short-term prediction task, our model achieved an AUC of 0.95, significantly outperforming traditional machine learning models and baseline deep learning models. Notably, at a clinically relevant high-specificity threshold, the model's true positive rate demonstrated a relative improvement of 23.6% over the next-best model, CNN.

As the prediction time window extends to 14 days, despite the increased predictive complexity, our model maintains a high AUC value (0.88), consistently outperforming alternative algorithms. Under the 28-day long-term prediction window, the model exhibits robust stability with an AUC of 0.91, whereas other methods such as CNN (0.86) and DNN (0.85) demonstrate comparatively inferior performance.

Importantly, across all time windows, traditional machine learning methods such as RandomForestClassifier and LogisticRegression consistently underperform compared to deep learning approaches, confirming the superiority of deep learning architectures in capturing complex temporal features within sepsis clinical data. Furthermore, the relatively minor performance fluctuation observed as prediction time windows extend indicates the temporal robustness of our approach, providing reliable decision support for early intervention in clinical practice. This characteristic enables our model to maintain high sensitivity while reducing long-term misdiagnosis risk, fulfilling the requirements for clinical dynamic monitoring.

### 3.2 Explainability analysis

The SHAP methodology, based on Shapley values from game theory, quantifies each feature's contribution to individual predictions, providing verifiable interpretations for deep learning models. This approach demonstrates strong alignment between influential features and clinical guidelines, enhancing model decision transparency and assisting clinicians in rapidly validating the medical rationale while optimizing individualized treatment strategies. SHAP values reflect the statistical associations between features and prediction outcomes, enabling clinicians to incorporate both direct causal effects and proxy variable effects during decision-making processes.

**Figure 5:**
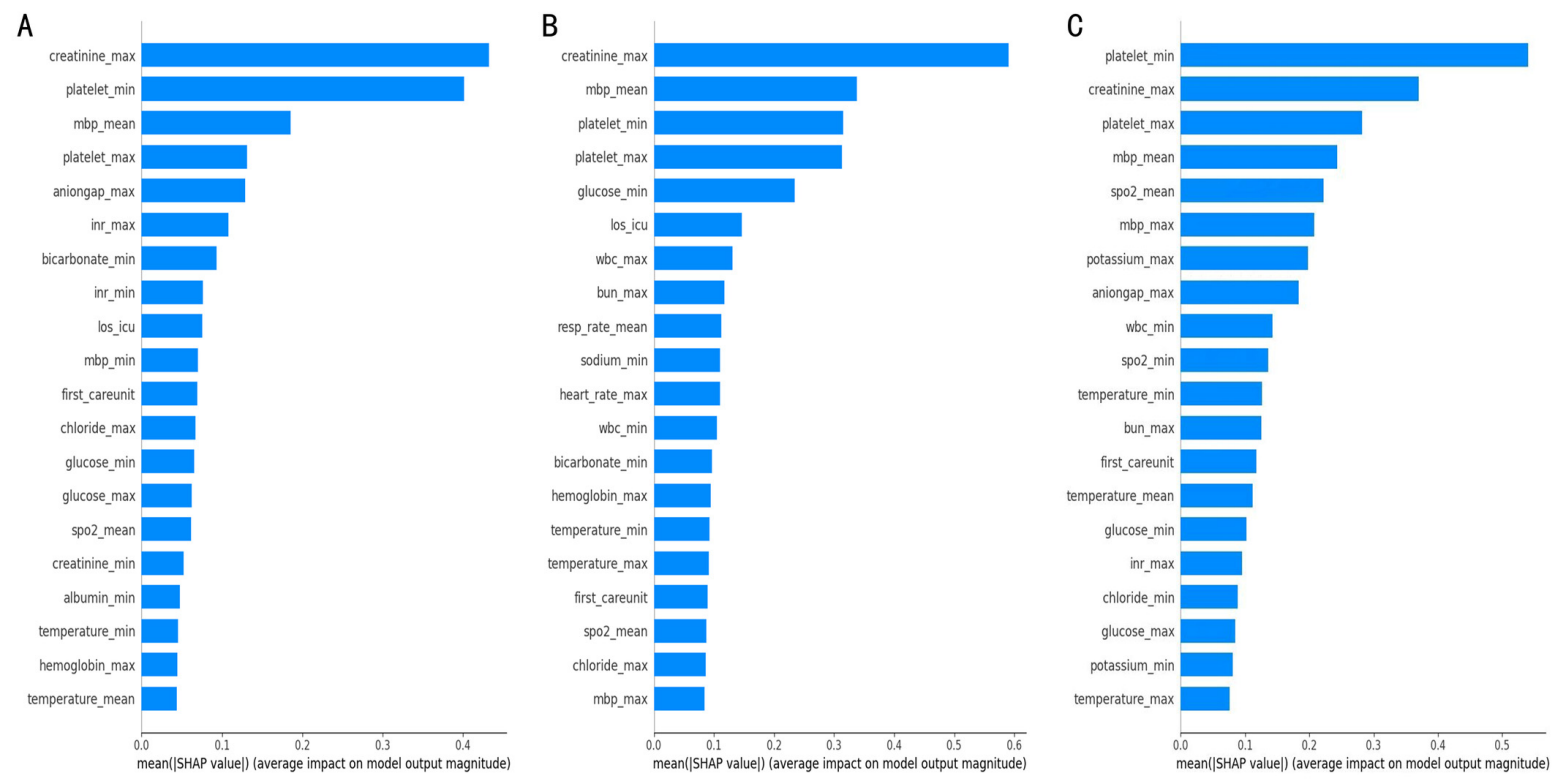
SHAP summary plot for the top 20 clinical features contributing to the model. **(A–C)**, respectively, represent independent cohorts sampled 7, 14, and 28 days prior to the onset of sepsis. Key predictive features influencing the model's output for each cohort were identified using SHAP value analysis.

**Figure 6:**
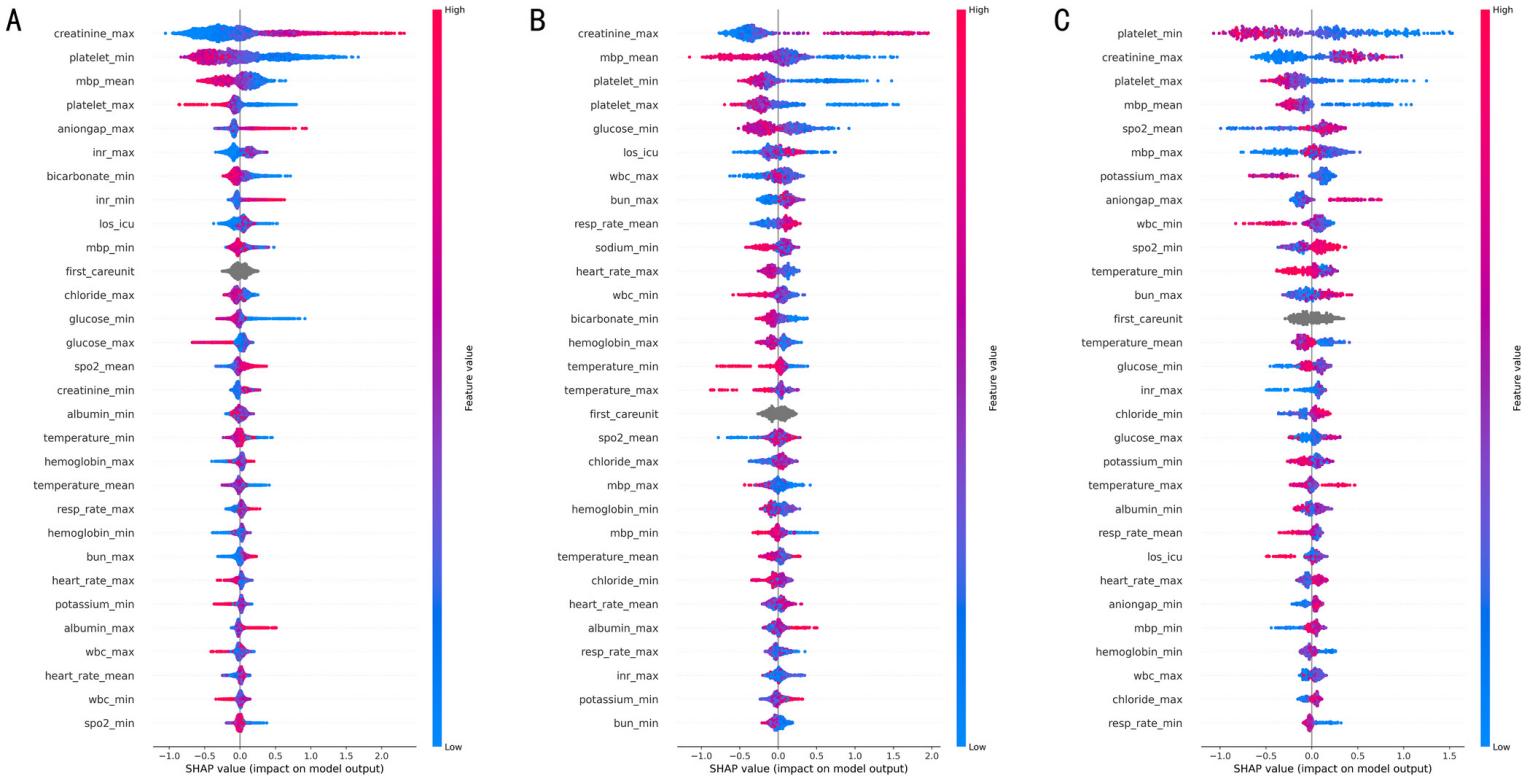
Beeswarm plots. **(A–C)** Present SHAP beeswarm plots for independent patient cohorts stratified by pre-onset intervals (7-, 14-, and 28-day groups). Within these plots, features are ranked in descending order of importance from top to bottom; the color of each point signifies the Feature value (red: high, blue: low), and the x-axis denotes the SHAP value. Negative SHapley values indicate that a given feature contributes to a lower predicted risk score, while positive Shapley values indicate a contribution to higher predicted risk.

The overall feature importance ranking and summary plot (Figure 5) clearly illustrate the global contribution and distribution of features across the dataset. Across all three prediction horizons, platelet_min (minimum platelet count) and creatinine_max (maximum creatinine level) consistently ranked as the most important features, exerting the most significant influence on the model's predictions. This indicates that the model effectively learned to prioritize variables critically for assessing renal and coagulation function, aligning with their fundamental roles in sepsis pathophysiology. At a more granular level, Figure 6 presents SHAP value distributions for each time window, detailing how variations in individual feature measurements impact the prediction score. Generally, lower platelet_min values and higher creatinine_max values increased the prediction score, thereby driving positive predictions (i.e., sepsis alerts). The high importance attributed to mbp_mean (mean arterial pressure) is consistent with clinical logic, as low MAP (< 65 mmHg) suggests shock and is associated with increased mortality risk.

Minimum glucose level demonstrated moderate importance (Figure 5), corroborating findings from related studies (54) that link hypoglycemia to potential sepsis presence. Although features like temperature_mean and hemoglobin_min had lower overall importance rankings, they still contributed to the model's predictions, reflecting the known association between factors like hemoglobin levels and sepsis risk. The collective influence of these features underpins the model's ability to predict sepsis onset.

The sepsis prediction model exhibits dynamic feature importance across different time windows, reflecting the model's sensitivity to disease progression. In the 7-day window (Figure 6A), maximum respiratory rate is associated with acute respiratory compensation, consistent with its role as an early warning indicator. Furthermore, SHAP contributions of features reflecting acid-base balance (e.g., anion gap, bicarbonate) and coagulation (International Normalized Ratio, INR) are significantly higher within this initial window compared to those in the 14- and 28-day horizons. This finding aligns with the acute physiological disturbances characteristic of early sepsis and underscores the model's ability to detect risks associated with acid-base imbalances.

Transitioning to the 14-day window (Figure 6B), features representing platelet count extremes exhibit markedly higher importance compared to the 7-day window. Similarly, the importance of minimum glucose level increases. As the time window extends to 14 days, the contribution of mean arterial pressure rises significantly. Beyond 14 days (Figure 6C), the relative importance of predictors appears more stable, although fluctuations in platelet count extremes remain highly correlated with sepsis progression, potentially suggesting significant platelet activation or pro-inflammatory states. This sensitivity to both high and low platelet extremes may offer greater diagnostic utility than traditional single-threshold monitoring. Notably, mean oxygen saturation shows significantly increased importance in the 28-day window, indicating that oxygenation status becomes a more critical predictive factor in long-term prediction. The 28-day feature importance distribution becomes more dispersed, suggesting that long-term prediction necessitates considering additional factors such as cumulative organ damage, altered immunometabolism, and iatrogenic risks. Despite this dispersion, indicators of renal function and hematological parameters remain crucial.

#### 3.2.1 Nonlinear interaction analysis

**Figure 7:**
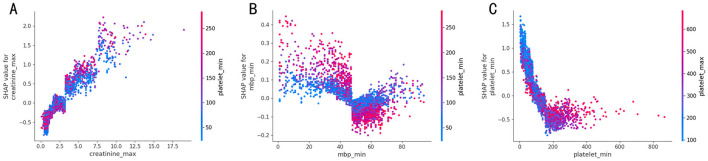
SHAP dependency plots for representative clinical features contributing to the model: maximum creatinine, minimum mean arterial pressure, and minimum platelet count. The panels display the impact of three key predictors: **(A)** Maximum creatinine, **(B)** Minimum mean arterial pressure, and **(C)** Minimum platelet count. Each point represents an individual sample. SHAP values greater than zero indicate an increased predicted risk of sepsis associated with the corresponding feature value. Point color, determined by the secondary (interaction) feature in each panel, visualizes interaction effects, showing how the secondary feature modulates the primary feature's contribution to the prediction.

SHAP dependence plot (Figure 7) illustrating how a primary feature's value influences its contribution to the model's prediction.

##### 3.2.1.1 Dual risk correlation

Figure 7A depicts the interaction between maximum creatinine and minimum platelet count in predicting sepsis risk. Maximum creatinine consistently ranks among the most influential predictors, potentially complementing other features for enhanced risk stratification. creatinine_max exhibits a strong positive correlation with SHAP values, indicating that higher levels consistently increase the predicted risk score, potentially reflecting early clinical manifestations of acute kidney injury (AKI).

When creatinine_max falls below a certain threshold, platelet count shows a significant negative correlation with SHAP values; specifically, lower platelet_min values (blue points) are associated with higher SHAP values (higher predicted risk). This observation aligns with a potential mechanism of concurrent platelet consumption and acute kidney injury during sepsis progression.

Conversely, in the higher creatinine range, a contrasting positive correlation is observed: some instances with high creatinine_max and concurrently elevated platelet_min are also associated with positive SHAP values, increasing the predicted risk. This complex pattern may reflect distinct underlying pathological states.

##### 3.2.1.2 Regulatory role of platelet count

Low mean arterial pressure (MAP) values (< 60 mmHg) were observed to correspond with high SHapley values, indicating their positive contribution to the model's prediction of sepsis risk. This finding aligns with the clinical understanding (55) of septic shock, where low MAP is recognized as a key indicator of adverse outcomes.

Figure 7B illustrates the interaction effect between minimum mean arterial pressure and minimum platelet count on model predictions. Specifically, when mbp_min decreases to values below 40 mmHg, SHAP values become predominantly positive, indicating that severe hypotensive states are associated with an elevated risk of adverse outcomes. This observation aligns with established clinical knowledge that sustained hypotension results in inadequate tissue perfusion. Interestingly, in the presence of relatively higher platelet_min values when mbp_min is low, these positive SHAP values become even larger, signifying an amplified predicted sepsis risk. Conversely, at higher mbp_min levels, SHAP values tend to become negative, suggesting these higher MAP values are associated with a lower predicted sepsis risk by the model.

The interaction between minimum platelet count and minimum mean arterial pressure exhibits a complex, non-monotonic relationship with the predicted sepsis risk. In the intermediate ranges, SHAP values exhibit significant fluctuations, with certain intervals showing positive values (indicating elevated risk) and others displaying negative values (suggesting risk reduction). In higher mbp_min ranges, elevated platelet_min values attenuate the predictive tendency of mbp_min toward sepsis risk, whereas, in lower mbp_min ranges, high platelet counts paradoxically intensify the predicted sepsis risk propensity as mbp_min decreases.

##### 3.2.1.3 Platelet dynamic trajectory analysis

Figure 7C illustrates the combined influence of the minimum platelet count and the maximum platelet count on the predicted sepsis risk, as quantified by SHAP values. When platelet_min falls below 100 × 10^9^/L (represented on the left portion of the x-axis), the corresponding SHAP values are positive and become increasingly positive as the count decreases further. This finding strongly aligns with the International Society on Thrombosis and Haemostasis (ISTH) diagnostic criteria for disseminated intravascular coagulation (DIC), which utilizes a platelet threshold of < 100 × 10^9^/L. Conversely, when the minimum platelet count is relatively high, the SHAP values tend to be slightly negative, indicating a modestly reduced predicted sepsis risk associated with higher baseline platelet levels.

Interestingly, a key observation arises in the low platelet_min range. SHAP values remain significantly positive (indicating high predicted risk) even when the corresponding maximum platelet count during the observation period was relatively high. This indicates that a significant reduction in platelets, resulting in a low platelet_min, serves as a critical indicator of increased sepsis risk, potentially offering greater predictive utility than the peak platelet count. Therefore, the dynamic variation in platelet count, reflected by the interplay between platelet_min and platelet_max, appears to capture predictive information beyond that available from static measurements alone.

### 3.3 Cross-dataset performance evaluation

Evaluating prediction models solely on a single dataset may constrain the assessment of their real-world applicability due to potential dataset-specific biases or overfitting. Multi-center data validation can mitigate performance degradation stemming from geographical or equipment variations; therefore, we evaluated our model on two additional datasets.

The datasets used for external validation were the eICU (56) Collaborative Research Database (eICU-CRD) and the Critical Care Database for Infected Patients from Zigong Fourth People's Hospital (57). The eICU-CRD, a multi-center intensive care unit (ICU) database developed by the Massachusetts Institute of Technology (MIT) in collaboration with Philips Healthcare, is designed to facilitate critical care research and computational algorithm development. It encompasses ICU patient data from over 200 hospitals across the United States, comprising approximately 200,000 ICU admission records from 2014 to 2015 across diverse ICU types. With its large scale and broad geographical representation, the database serves as a valuable complementary resource to other critical care databases like MIMIC.

The second dataset is the Critical care database comprising patients with infection at Zigong Fourth People's Hospital (v1.1) (57) (hereafter, “*Zigong*” dataset), reportedly the first publicly available multi-source clinical database focusing on adult ICU patients with infections in China. It was developed through collaboration between the Zigong Fourth People's Hospital in Sichuan province and multiple research institutions. This database includes data from infected patients admitted to the hospital's ICU between 2019 and 2020. The database features comprehensive data integration and incorporates specialized procedures for keyword extraction and cleaning of clinical notes originally recorded in Chinese. This resource addresses a significant gap in publicly available Asian critical care infection data, thereby contributing to global critical care research and facilitating the adaptation and validation of AI-based medical tools for diverse populations.

The researchers obtained approval from the relevant institutional review board (IRB) and were granted permission to access and utilize the database for data extraction and analysis in accordance with established protocols. As detailed in Table 3, our proposed model demonstrated robust generalization capabilities when evaluated on two independent and heterogeneous clinical datasets (58): the *Zigong* dataset and the eICU database. It achieved Area Under the Receiver Operating Characteristic Curve (AUC) values of 0.978 ± 0.074 and 0.982 ± 0.090 on the *Zigong* and eICU datasets, respectively. This performance significantly surpasses that of the benchmark traditional machine learning and deep learning models, exceeding the highest performing baseline (RandomForest-Classifier on eICU) by 3.6% points and the lowest (LogisticRegression on *Zigong*) by 8.6% points. Notably, the model maintained a strong balance between precision and recall, achieving F1-scores of 0.946 ± 0.078 on the *Zigong* dataset and 0.951 on the eICU dataset. Compared to XGBoost, this represents an improvement of 3.4% points on *Zigong* and 2.7% points on eICU. Against LSTM, the improvements were 7.1% points on *Zigong* Data and 6.4% points on eICU. This consistent performance across diverse datasets underscores the model's robustness and generalizability to variations in data collection protocols and clinical environments.

**Table 3:** Comparison results of the performance of the model with different models in other clinical data.

**Dataset**	**Models**	**AUC**	**Accuracy**	**Precision**	**Recall**	**F1-score**
Zigong	Our model	0.978 ± 0.074	0.945 ± 0.047	0.952 ± 0.023	0.940 ± 0.042	0.946 ± 0.078
	CNN	0.923 ± 0.016	0.892 ± 0.041	0.885 ± 0.048	0.898 ± 0.060	0.891 ± 0.085
	DNN	0.915 ± 0.003	0.887 ± 0.031	0.879 ± 0.076	0.895 ± 0.017	0.887 ± 0.053
	LSTM	0.908 ± 0.070	0.875 ± 0.060	0.868 ± 0.028	0.882 ± 0.026	0.875 ± 0.052
	XGBClassifier	0.941 ± 0.028	0.912 ± 0.049	0.920 ± 0.052	0.905 ± 0.021	0.912 ± 0.088
	RandomForestClassifier	0.934 ± 0.022	0.907 ± 0.063	0.915 ± 0.059	0.900 ± 0.016	0.907 ± 0.027
	MLPClassifier	0.918 ± 0.100	0.890 ± 0.094	0.883 ± 0.006	0.897 ± 0.054	0.890 ± 0.084
	LogisticRegression	0.892 ± 0.050	0.853 ± 0.085	0.847 ± 0.033	0.859 ± 0.052	0.853 ± 0.005
	SVC	0.901 ± 0.089	0.868 ± 0.002	0.861 ± 0.026	0.875 ± 0.038	0.868 ± 0.042
eICU	Our model	0.982 ± 0.090	0.951 ± 0.037	0.958 ± 0.063	0.944 ± 0.001	0.951 ± 0.066
	CNN	0.915 ± 0.067	0.901 ± 0.045	0.910 ± 0.079	0.892 ± 0.010	0.901 ± 0.030
	DNN	0.927 ± 0.027	0.894 ± 0.077	0.903 ± 0.059	0.885 ± 0.077	0.894 ± 0.030
	LSTM	0.920 ± 0.020	0.887 ± 0.081	0.896 ± 0.085	0.878 ± 0.010	0.887 ± 0.052
	XGBClassifier	0.903 ± 0.042	0.894 ± 0.062	0.900 ± 0.014	0.889 ± 0.070	0.924 ± 0.012
	RandomForestClassifier	0.946 ± 0.050	0.917 ± 0.032	0.925 ± 0.060	0.909 ± 0.004	0.917 ± 0.000
	MLPClassifier	0.930 ± 0.044	0.897 ± 0.073	0.906 ± 0.059	0.888 ± 0.084	0.897 ± 0.028
	LogisticRegression	0.898 ± 0.021	0.857 ± 0.090	0.851 ± 0.093	0.863 ± 0.001	0.857 ± 0.073
	SVC	0.913 ± 0.076	0.879 ± 0.010	0.872 ± 0.045	0.886 ± 0.053	0.879 ± 0.058

## 4 Discussion

In this study, we propose a novel deep-learning framework for the early prediction of sepsis, specifically targeting detection within a clinically actionable timeframe of 7–28 days prior to the onset of clinical suspicion. Our approach uniquely integrates structured state-space models (SSMs) within a Boosting framework. This novel integration facilitates the automatic adjustment of feature weight distributions, enables the construction of dynamic surrogate models through a forward-backward optimization mechanism, and enhances computational efficiency and scalability via multi-paradigm integration. Consequently, our framework aims to overcome limitations inherent in traditional single-paradigm predictive models. Experimental results demonstrate that our model achieves consistently high predictive accuracy across the defined time windows. Additionally, the framework supports model interpretability through the application of SHAP (SHapley Additive exPlanations) values for assessing local feature contributions and overall feature importance analysis.

Across various public Electronic Health Record (EHR) datasets, our model achieved a predictive accuracy ranging from 0.978 ± 0.074 to 0.982 ± 0.090 across diverse external datasets, demonstrating superior performance compared to baseline deep learning and traditional machine learning methods. This validates the effectiveness, generalizability, and efficiency of our model. It can rapidly adapt to diverse clinical settings, thereby paving the way for responsive healthcare systems.

Early diagnosis of sepsis currently faces numerous challenges. While approaches such as genomic profiling, monitoring of inflammatory markers, and EHR-based machine learning models have been proposed, patient heterogeneity, data noise, and variations in clinical workflows substantially impair their performance. Although current clinical diagnostic methods, such as the Sequential Organ Failure Assessment (SOFA) score and Procalcitonin/C-reactive Protein (PCT/CRP) testing, are widely adopted, their static assessment approach struggles to capture the dynamic pathological progression (AUC: 0.61–0.88) (59), and they fail to detect early baseline fluctuation signals during the initial stages of infection (59). The lightweight dynamic prediction model developed in this study enables continuous monitoring within the 7- to 28-day window prior to clinical suspicion, significantly extending the intervention window compared to traditional methods (predictive accuracy: 0.978–0.982). Furthermore, its adaptability across multiple time windows and strong generalizability provides critical infrastructure support for regional sepsis surveillance.

The model is strategically designed to meet the diverse requirements of various medical scenarios. Its lightweight architecture reduces computational demands, enabling operation on standard CPUs. Furthermore, its dynamic multi-paradigm integration mechanism facilitates optimal resource allocation, while the modular design allows for rapid implementation in primary healthcare settings. In contrast to existing treatment-oriented systems (60), the proposed model addresses limitations inherent in traditional algorithms for high-dimensional feature selection through a forward-backward optimization process and a dynamic agent framework. Additionally, SHAP values are employed to enhance clinical interpretability by identifying key predictors, such as creatinine levels, thereby supporting informed decision-making and fostering clinical trust. Economic analyses (61) indicate that medical costs for sepsis patients significantly exceed those for non-infected individuals, particularly when associated with nosocomial infections, where costs can increase substantially. Consequently, early warning facilitated by such models holds the potential to reduce complication rates and mortality, thereby alleviating the overall healthcare burden.

Nevertheless, this study has certain limitations: the heterogeneity of public databases could result in model generalization bias; while the sample imbalance issue was mitigated through the use of SMOTE, synthetic data could introduce clinically irrelevant artifacts; moreover, unmeasured confounding variables, including race and treatment preferences, as well as the generalizability to pediatric populations, require further validation through multi-center studies.

Future research will prioritize several key areas. These include leveraging Hospital Information Systems (HIS) for embedded Application Programming Interface (API) development, establishing a closed-loop management system encompassing the continuum from diagnosis to intervention, and optimizing individualized prediction accuracy via integrated multi-source data. Such advancements aim for healthcare professionals to rapidly access critical patient indicators and perform effective risk stratification. Ultimately, this work seeks to improve the overall quality of sepsis care—spanning diagnosis, treatment, and management—while enhancing the efficiency of resource utilization.

## 5 Conclusion

In summary, our proposed BOBM model, through a deeply coupled framework of Mamba-Tab and Boosting techniques, effectively enables early sepsis prediction across various time windows. Its lightweight architecture facilitates clinical deployment, and it demonstrates strong generalization capabilities. These innovations enhance the utility of clinical physiological indicators as predictive features, while simultaneously improving model interpretability.

## Data Availability

The datasets presented in this study are publicly available. The MIMIC-IV dataset can be found on PhysioNet (https://physionet.org/content/mimiciv/). The eICU-CRD is available at https://physionet.org/content/eicu-crd/.
