# Supplementary material for: BOBM: an adaptive deep learning framework for extended-window sepsis prediction with cross-institutional generalizability
Source: Front Med (Lausanne). 2026 Jan 15;12:1731854. doi: 10.3389/fmed.2025.1731854 (PMC12852446; doi:10.3389/fmed.2025.1731854)
Supplement: Supplementary file 1 [file Table_1.docx]

## Supplementary Methods

## 1. Mamba tab

Mamba is a linear time-series modeling approach (1) centered on the Selective State Space Model. A key innovation lies in dynamizing the State Space Model (SSM) parameters, formulating them as functions of the input. This enables the model to selectively propagate or forget information along the sequence dimension according to the specific input (2). Mamba Tab is an extension of Mamba, specifically optimized for regression tasks involving tabular data. It integrates the principles of gating mechanisms, thereby enhancing feature selection and controlling information flow. This contributes to its superior performance in tabular data modeling.

**1.1 Enhanced sequence modeling**

Mamba enhances sequence modeling capabilities by employing State Space Models (SSM) to represent input sequences (e.g., table rows or columns) as Linear Time-Invariant (LTI) systems. Information is transmitted via hidden states, overcoming the efficiency bottlenecks inherent in traditional RNNs and Transformers when processing long sequences.

**1.2 Improved adaptation to tabular data**

Mamba Tab treats each row of a table as an independent sequence or transforms columns into sequences via feature embedding, thereby inheriting Mamba’s ability to capture long-range dependencies. In the data preprocessing phase, input data encompassing numerical and categorical features are processed. Numerical features are normalized via Min-Max Scaling to scale values within the [0,1] range; categorical features are encoded using Ordinal Encoding to enable the model to process both numerical and categorical features concurrently, thereby ensuring dimensional consistency of embeddings in incremental learning scenarios (2). The embedding learning module takes the preprocessed features as input and employs a fully connected layer to learn low-dimensional dense vector embeddings (2). This embedding process directly learns multi-dimensional representations from the input features, thereby mitigating the influence of arbitrary feature ordering on model performance and furnishing standardized inputs for the subsequent Mamba Tab module. Mamba Tab employs modules such as linear projection, convolution, and dynamic state modeling to extract and model complex dependencies in tabular data.

Initially, the input features are processed through two separate branches for linear projection, resulting in two feature subspace representations (2):

| $z_{1}=LP_{1}(u), z_{2}=LP_{2}(u)$ | (1) |
| --- | --- |

Where denotes the input feature sequence (with length *T* and feature dimension *d*), and *LP*_1_ and *LP*_2_ are independent linear transformations. Subsequently, *z*_1_ undergoes one-dimensional causal convolution to extract local features and short-range dependencies. The output of the convolution operation is subsequently fed into the State Space Model (SSM) to model long-range dependencies. This model characterizes feature dynamics using the following continuous-time dynamic equations (3):

| $\frac{dh\left(t\right)}{dt}=Ah\left(t\right)+Bu\left(t\right),x\left(t\right)=Ch\left(t\right)$ | (2) |
| --- | --- |

$h(t)$: the hidden state vector at continuous time *t*. $u(t)$: the input feature sequence. $x(t)$: the output feature representation.

A, B, and C are the state transition matrix, input projection matrix, and output projection matrix, respectively.

For application to discrete data, the state-space model is subsequently discretized into the following form:

| $h_{k}=\overline{A}h_{k-1}+\overline{B}u_{k},x_{k}=Ch_{k}$ | (3) |
| --- | --- |
| Where *h_k_* and u_k_ denote the hidden state and input at discrete time step *k*, respectively.  The discretized state transition matrices are given by |  |
| $\overline{A}=\exp\left(\Delta A\right),\overline{B}=\left(\Delta A\right)^{-1}\left(\exp\left(\Delta A\right)-I\right)B$ | (4) |

Δ: the discrete-time step.

These matrices are input-dependent functions, which allow the SSM model to dynamically and selectively propagate or forget information, thereby adapting to the requirements of different contexts.

The output of the SSM undergoes a nonlinear transformation via the *SiLU* activation function:

| $SiLU\left(x\right)=x\cdot\sigma\left(x\right),\sigma\left(x\right)=\frac{1}{1+\exp\left(-x\right)}$ | (5) |
| --- | --- |

The activated result is multiplied by the linear projection *LP*2(*u*) from the second branch, further enhancing the model’s feature selection capability. The activated output is element-wise multiplied by the linear projection *LP*2(*u*) from the second branch, further enhancing the model’s feature selection capabilities (4). The Mamba Tab module can be stacked across multiple layers, enabling effective information propagation through residual connections to mitigate the vanishing gradient problem.

The final output from the Mamba Tab module is projected onto the target output space using a fully connected layer for prediction. For classification tasks, the final model output is transformed into a probability score using the Sigmoid activation function, as described by the formula :

| $\widehat{y_{i}}=Sigmoid\left(FC\left(x\right)\right)$ | (6) |
| --- | --- |

where:

$\widehat{y_{i}}$ : denotes the predicted probability for the *i*-th sample.

*FC*: represents the fully connected layer that transforms high-dimensional features into a solitary output value.

*x*: represents the output feature vector from the preceding Mamba Tab module.

### 2. Boosting

Boosting, an ensemble learning methodology, sequentially integrates a collection of base predictors (often termed weak learners), ultimately yielding a model with robust predictive power. Fundamentally, Boosting utilizes reweighted training data within an iterative optimization framework to enhance the classifier’s performance, particularly in difficult-to-classify instances (5).

Let the training set be denoted by , where *x_i_* represents the input feature vector and *y_i_* ∈ {–1, +1} is the corresponding binary class label. And *N* is the number of training samples.

Initially, all training samples are assigned uniform weights, typically defined as:

|  | (7) |
| --- | --- |

is the weight of sample *i* at iteration *t. t=1, …, T,* where *T* is the total number of boosting rounds.

The Boosting process proceeds iteratively. In the *t*-th iteration:

During the *t*-th iteration, a weak learner $h_{t}(x)$is trained using the current sample weight distribution $w_{t}$. The weighted error rate of $h_{t}(x)$ is calculated:


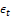

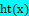


|  | (8) |
| --- | --- |

where is the indicator function (1 if the condition is true, 0 otherwise) (6).


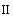

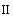


Subsequently, *A* weight is assigned to the weak learner, quantifying its contribution:


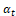


|  | (9) |
| --- | --- |

Here, $\ln(\cdot)$ denotes the natural logarithm. Notably, > 0 if the learner’s error  is less than 0.5, signifying a positive contribution to the final ensemble.


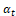

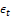


The sample weights $w_{t+1}\left(i\right)$ are then updated based on the performance of the weak learner. Given the fixed learner weight $\alpha_{t}$, the weight of each training example is updated as:

| $w_{t+1}\left(i\right)=w_{t}\left(i\right)\cdot\exp\left(-\alpha_{t}\cdot y_{i}\cdot h_{t}\left(x_{i}\right)\right)$ | (10) |
| --- | --- |

So that misclassified samples (for which $y_{i}\neq h_{t}(x_{i})$ ) receive a multiplicative increase in their weights, whereas correctly classified samples receive a multiplicative decrease. Here, $w_{t}(i)$ denotes the weight of the *i*-th training example at iteration *t*, and $\alpha_{t}$denotes the learner weight derived from the error rate (as calculated in Eq. 9).

After normalization, the resulting weights form the distribution used for training the weak learner in the subsequent iteration.

This iterative process of training weak learners and updating weights continues until a predefined number of iterations (*T*) is reached or another stopping criterion is met.

Finally, the overall strong classifier *H*(*x*) is constructed by aggregating the predictions of all *T* weak learners via a weighted majority vote, as shown in equation:

| $H\left(x\right)=sign\left(\sum_{t=1}^{T} \alpha_{t}\cdot h_{t}\left(x\right)\right)$ | (11) |
| --- | --- |

### 3 Bayesian optimization

Bayesian Optimization (BO) offers an efficient framework for optimizing expensive black-box functions, particularly when analytical forms are unavailable (7). This approach constructs probabilistic surrogate models of the objective

function, typically using Gaussian processes, and employs acquisition functions to balance exploration and exploitation while seeking the global optimum with minimal function evaluations (8, 9).

**3.1 Mathematical Model**

Our objective is to identify the global optimum over a defined domain *x*:

| $x^{*}=\arg\max_{x\in X} f\left(x\right)$ | (12) |
| --- | --- |

where$f\left(x\right)$denotes the black-box objective function to be optimized.

**3.2 Core Components**

(1) Gaussian Process Model

Given a dataset $\text{D}_{\text{n}}=\left\{\left(\text{x}_{\text{i}},\text{y}_{\text{i}}\right)\right\}_{\text{i}=1}^{\text{n}}$comprising n sampling points:

|  | (13) |
| --- | --- |

where $v_{i}$ is independent Gaussian noise with variance $\sigma_{z}^{2}$.

The Gaussian process provides a posterior distribution over the objective function:

| $f\left(x\right)\sim N\left(\mu\left(x\right),\sigma^{2}\left(x\right)\right)$ | (14) |
| --- | --- |

where:

| $\mu\left(x\right)=K\left(x,X\right)\left[K\left(X,X\right)+\sigma_{z}^{2}I\right]^{-1}y$ | (15) |
| --- | --- |
| $\text{σ}^{2}\left(x\right)=k\left(x,x\right)-K\left(x,X\right)\left[K\left(X,X\right)\text{+ σ}_{z}^{2}\text{I}\right]^{-1}K\left(x,X\right)^{\top}$ | (16) |

In the above expressions, $k(\cdot,\cdot)$ denotes the kernel function. is the kernel matrix with entries $[K(X,X)]_{ij}=k(x_{i},x_{j})$ , and is the row vector of kernel values between the test point *x* and all training points, i.e., $K(x,X)=[k(x,x_{1}),\ldots,k(x,x_{n})]$. *I* is the $n\times n$ identity matrix and $\sigma_{z}^{2}$ is the noise variance.

The kernel function typically employs the Radial Basis Function (RBF) kernel:

| $k(x,x')=\sigma_{f}^{2}\exp\left(-\frac{\text{‖}x-x'\text{‖}^{2}}{2l^{2}}\right)$ | (17) |
| --- | --- |

where $\sigma_{f}^{2}$ is the signal variance and *l* is the length-scale hyperparameter.

(2) Acquisition Functions

Acquisition functions guide sampling point selection by balancing exploration and exploitation:

Expected Improvement (EI):

| $\begin{matrix} EI\left(x\right)=\left(\mu\left(x\right)-f^{*}-\xi\right)\Phi\left(z\right)+\sigma\left(x\right)\emptyset\left(z\right) \\ \text{ }z=\frac{\mu(x)-f^{*}-\xi}{\sigma(x)}, \end{matrix}$ | (18) |
| --- | --- |

where $f^{*}$ denotes the current best observed objective value, *z*, $\mu(x)$ and $\sigma(x)$ are the predictive mean and standard deviation from the Gaussian process model, and $\Phi(\cdot)$ and are the cumulative distribution function and probability density function of the standard normal distribution (10), respectively. The parameter $\xi$ controls the exploration–exploitation trade-off.

Upper Confidence Bound (UCB):

| $UCB\left(x\right)=\mu\left(x\right)+\beta\sigma\left(x\right)$ | (19) |
| --- | --- |

where *β* is a hyperparameter that controls the exploration-exploitation balance.

(3) Bayesian Optimization Procedure

Initialization: Select an initial set of points. Fit an initial Gaussian Process (GP) model to the initial dataset.

Sequential selection: Select the next sampling point by maximizing the acquisition function:

| $x_{n+1}=\arg\max_{x\in X} \alpha\left(x;D_{n}\right)$ | (20) |
| --- | --- |

where is the acquisition function (e.g., EI or UCB) computed using the current dataset .

Evaluate the objective function and update the dataset.

| $\text{y}_{\text{n}+1}=\text{f}\left(\text{x}_{\text{n}+1}\right)+\text{ε}$ | (21) |
| --- | --- |
| $\text{D}_{\text{n}+1}=\text{D}_{\text{n}}\cup\left\{\left(\text{x}_{\text{n}+1},\text{y}_{\text{n}+1}\right)\right\}$ | (22) |

Model update: Update the Gaussian process model. Repeat steps 2-3 until termination criteria are met (e.g., computational budget or convergence).

**Supplemental Table S1:** List of extracted clinical variables.

| **Serial number** | **Feature** | **Description** |
| --- | --- | --- |
| 1 | admission age | Age (years) |
| 2 | los | Length of stay in ICU |
| 3 | icu | Intensive care unit |
| 4 | sofa | Sequential organ failure assessment |
| 5 | heart_rate_min | Minimum heart rate (beats per minute) |
| 6 | heart_rate_max | Minimum heart rate (beats per minute) |
| 7 | heart_rate_mean | Mean heart rate (beats per minute) |
| 8 | mbp_min | Minimum mean arterial pressure (mm Hg) |
| 9 | mbp_max | Maximum mean arterial pressure (mm Hg) |
| 10 | mbp_mean | Maximum mean arterial pressure (mm Hg) |
| 11 | resp_rate_min | Minimum respiration rate (breaths per minute) |
| 12 | resp_rate_max | Maximum respiration rate (breaths per minute) |
| 13 | resp_rate_mean | Mean respiration rate (breaths per minute) |
| 14 | temperature_min | Minimum temperature (°C) |
| 15 | temperature_max | Maximum temperature (°C) |
| 16 | temperature_mean | Mean temperature (°C) |
| 17 | spo2_min | Minimum pulse oximetry (%) |
| 18 | spo2_max | Maximum pulse oximetry (%) |
| 19 | glucose_min | Minimum serum glucose (mg/dL) |
| 20 | glucose_max | Maximum serum glucose (mg/dL) |
| 21 | weight_min | Minimum weight (kg) |
| 22 | weight_max | Maximum weight (kg) |
| 23 | hemoglobin_min | Minimum hemoglobin (g/dL) |
| 24 | hemoglobin_max | Maximum hemoglobin (g/dL) |
| 25 | platelet_min | Minimum platelet count (K/uL or 10^9^/L) |
| 26 | platelet_max | Maximum platelet count (K/uL or 10^9^/L) |
| 27 | wbc_min | Minimum white blood cell count (K/uL or 10^9^/L) |
| 28 | wbc_max | Maximum white blood cell count (K/uL or 10^9^/L) |
| 29 | albumin_min | Minimum albumin (g/dL) |
| 30 | albumin_max | Maximum albumin (g/dL) |
| 31 | anion_gap_min | Minimum anion gap (mEq/L) |
| 32 | anion_gap_max | Maximum anion gap (mEq/L) |
| 33 | bicarbonate_min | Minimum bicarbonate (mEq/L) |
| 34 | bicarbonate_max | Maximum bicarbonate (mEq/L) |
| 35 | bun_min | Minimum blood urea nitrogen (mg/dL) |
| 36 | bun_max | Maximum blood urea nitrogen (mg/dL) |
| 37 | chloride_min | Minimum chloride (mEq/L) |
| 38 | chloride_max | Maximum chloride (mEq/L) |
| 39 | creatinine_min | Minimum creatinine (mg/dL) |
| 40 | creatinine_max | Maximum creatinine (mg/dL) |
| 41 | sodium_min | Minimum sodium (mEq/L) |
| 42 | sodium_max | Maximum sodium (mEq/L) |
| 43 | potassium_min | Minimum potassium (mEq/L) |
| 44 | potassium_max | Maximum potassium (mEq/L) |
| 45 | inr_min | Minimum International Normalized Ratio/Minimum INR |
| 46 | inr_max | Maximum International Normalized Ratio/Maximum INR |
| 47 | lactate_min | Minimum lactate (mmol/L) |
| 48 | lactate_max | Maximum lactate (mmol/L) |
| 49 | urine_output | Urine output (mL or mL/hr) |

# References

1. He X, Cao K, Zhang J, Yan K, Wang Y, Li R, et al. Pan-Mamba: effective pan-sharpening with state space model. *Inf Fusion*. (2025) 115:102779. doi: 10.1016/j.inffus.2024.102779
2. Ahamed MA, Cheng Q. MambaTab: a plug-and-play model for learning tabular data. In: Proceedings of the 2024 IEEE 7th International Conference on Multimedia Information Processing and Retrieval (MIPR), San Jose, CA, USA. 2024, pp. 369–375. doi: 10.1109/MIPR62202.2024.00065
3. Gu A, Dao T. Mamba: linear-time sequence modeling with selective state spaces. arXiv preprint [Preprint] (2023). Available at: https://doi.org/10.48550/arXiv.2312.00752 (Accessed August 14, 2025).
4. Elfwing S, Uchibe E, Doya K. Sigmoid-weighted linear units for neural network function approximation in reinforcement learning. *Neural Netw.* (2018) 107:3–11. doi: 10.1016/j.neunet.2017.12.012
5. Freund Y, Iyer R, Schapire RE, Singer Y. An efficient boosting algorithm for combining preferences. *J Mach Learn Res.* (2003) 4:933–69. doi: 10.1162/1532443041827916
6. Bickel PJ, Ritov Y, Zakai A. Some theory for generalized boosting algorithms. *J Mach Learn Res.* (2006) 7:705–32. doi: 10.5555/1248547.1248572
7. Frazier P. A tutorial on Bayesian optimization. arXiv preprint [Preprint] (2018). Available at: https://doi.org/10.48550/arXiv.1807.02811 (Accessed August 14, 2025).
8. Di Fiore F, Nardelli M, Mainini L. Active learning and Bayesian optimization: a unified perspective to learn with a goal. *Arch Comput Methods Eng.* (2024) 31(5):2985–3013. doi: 10.1007/s11831-024-10064-z
9. Yu M, Yang S, Wu C, Marom N. Machine learning the Hubbard *U* parameter in DFT+*U* using Bayesian optimization. *npj* *Comput Mater.* (2020) 6:180. doi: 10.1038/s41524-020-00446-9
10. Wang X, Jin Y, Schmitt S, Olhofer M. Recent advances in Bayesian optimization. *ACM Comput Surv.* (2013) 55(13s):1–36. doi: 10.1145/3582078
